# Exposure of human lymphoma cells (U-937) to the action of a single mycotoxin as well as in mixtures with the potential protectors 24-epibrassinolide and selenium ions

**DOI:** 10.1007/s12550-018-0334-1

**Published:** 2018-11-08

**Authors:** Anna Barbasz, Elżbieta Rudolphi-Skórska, Maria Filek, Anna Janeczko

**Affiliations:** 1 Institute of Biology, Pedagogical University of Cracow, Podchorążych 2, 30-084 Cracow, Poland; 2 Institute of Plant Physiology, Polish Academy of Sciences, Podłużna 3, 30-239 Cracow, Poland

**Keywords:** Zearalenone, Mycotoxin, Human cells, Monocytes, Membranes, Langmuir monolayers, U-937

## Abstract

The progressive contamination of food products by mycotoxins such as zearalenone (ZEN) has prompted the search for specific substances that can act as protectors against an accumulation of these toxins. This paper discusses the effect of selenium ions and 24-epibrassinolide (EBR) as non-organic and organic compounds that preserve human lymphoblastic cells U-937 under ZEN stressogenic conditions. Based on measurements of cell viability and a DAPI test, concentrations of ZEN at 30 μmol/l, Se at 2.5 μmol/l and EBR at 0.005 μmol/l were selected. The addition of both protectors resulted in an increase in the viability of ZEN-treated cells by about 16%. This effect was connected with a decrease in lipid peroxidation (a decrease in the malonyldialdehyde content) and the generation of reactive oxygen species, which were determined by a cellular ROS/superoxide detection assay and the SOD activity. The Se protection was observed as the blocking of the all excess ROS, while the EBR action was mainly concentrated on something other than the superoxide radical itself. The experiments on the model lipid membranes that mimic the environment of U-937 cells confirmed the affect of ZEN on the structure and physicochemical properties of human membranes. Although the presence of both Se and EBR reduced the effect of ZEN by blocking its interaction with a membrane, the action of Se was more evident.

## Introduction

The contamination of plants, especially cereals, which serve as a food source for animals and humans, by mycotoxins is a global problem. Detoxification of plants is difficult because mycotoxins can be absorbed by crops during all of their growth phases and even after harvesting (Kumar et al. 2008). Mycotoxins, which are taken up together with plant nutrients, are then accumulated in the cells of animals and people, which pose serious hazards to their health (Negedu et al. 2011). The expression of their toxicity is regulated by factors such as age, sex, species and the status of the nutrition of contaminated animals (Hook and Williams 2004). Laboratory observations that have been performed on mice, rats, guinea pigs, hamsters and rabbits have shown that the effects of toxins are associated with many physiological changes in their organs, which can even lead to developing cancer (Belhassen et al. 2015; Creppy 2002; Osweiler 2000). It was found that the liver and kidneys were the predominant tumour sites (Banu et al. 2009; Bray and Ryan 1991). Other studies have indicated that there are additional toxic effects that are connected with immunity suppression, dysfunctions of mitochondrial DNA and the induction of anaemia, which are a consequence of a reduced oxygen supply to tissues (Akande et al. 2006; Aydin et al., 2007; Verma et al., 2004). Moreover, it was suggested that these substances may also affect the structure of the central nervous system (Laag and Elaziz 2013).

One of the mycotoxins that is currently being studied intensively is zearalenone (ZEN, F-2 toxin), which is produced by several species that infect *Fusarium*, especially cereals such as wheat, oats and barley, which are still the most common food crops in the world (Zinedine et al. 2007). Studies of the mechanism of ZEN action, which have been conducted in in vitro conditions for both animal and human cells, have shown that the effects of its cytotoxicity were mainly due to oxidative stress, which initiated lipid peroxidation (Gautier et al. 2001) and/or oxidative damage of DNA (Verma et al., 2004; Hassen et al. 2007; Tatay et al. 2016). Lipid degradation alters the structure and function of the cellular membrane and inhibits cellular metabolism, which leads to cytotoxicity. The inhibition of protein and DNA syntheses results in the perturbation of the progression of the cell cycle (Abid-Essefi et al. 2004), which induces DNA fragmentation and micronuclei production (Ouanes et al. 2003) and, as a result, leads to apoptosis. However, as was described by Abid-Essefi et al. (2004), the cytotoxicity of *Fusarium* toxins on epithelium cell lines is concentration- and time-dependent**.**

Many strategies can be undertaken to reduce the accumulation of mycotoxins in both crops and farm animals, including the use of absorbent materials, which may bind mycotoxins (Wageha et al. 2010) or supplementation with chemicals in order to reduce the stress-inducing effects of ZEN, e.g. quercetin (Escriva et al. 2017). In our earlier studies, we observed that selenium ions and brassinosteroids (24-epibrassinolide; EBR) may serve as protectors that diminish the uptake of ZEN by plant cells (Filek et al. 2017; Kornaś et al. 2018). Plant supplementation with Se has mainly been studied in respect to providing protection against heavy metal stresses. It was suggested that the defence against antioxidative damage of cells is involved in the mechanism of its action (Sieprawska et al. 2015). For mammalian cells, this microelement was indicated as an inhibitor of tumour cell growth in many studies (Batist et al. 1986; Spyrou et al. 1996; Stewart et al. 1997). Brassinosteroids (BRs) have also been examined as potential anticancer and antioxidative factors. It was shown that when used at micromolar concentrations, natural BRs can inhibit the growth of human cancer cell lines (Malíková et al. 2008) and reduce the levels of intracellular reactive oxygen species (Carange et al. 2011).

In the presented experiments, the human cell line U-937 was examined to investigate its potential Se/EBR effects against ZEN stress. A stable cell line enables observations of monocyte cell behaviour in vitro and has been used as a model of the cytotoxicity of a substance against the human immune system in many studies (Gomez et al. 1993; Park et al. 2002; Barbasz et al. 2017). Because they are widely distributed cells that are present in the blood and tissues, they come into contact with “foreign” substances such as, for example, xenobiotics like mycotoxins. This interaction should promote and modulate cells in the course of an immune response. The possibility of using both protectants against ZEN-stress in U-937 cells was demonstrated by analyses of the differences in: (i) the destruction of cell viability, (ii) the generation of cellular ROS/superoxide radicals, (iii) the stimulation of the antioxidative enzymes and (iv) the modification of membrane structures. Changes in the membrane properties that resulted from lipid oxidation were examined as an increase in the MDA concentration, which is generally considered to be an index of ROS membrane degradation (Tomita et al., 1990). Moreover, in the model membranes, which were built from the lipids that were present in the largest quantities in the studied cells, the specific interactions of the tested substances were also considered.

## Materials and methods

### Chemicals

The ZEN was obtained from Fermentek (Jerusalem, Israel). The 2,4-dinitrophenylhydrazine, 24-epibrassinolide (24-epibrassinolide, (22R, 23R, 24R)-2α,3α,22,23-tetrahydroxy-24-methyl-B-homo-7-oxa-5α-cholestane-6-one), 3-(4,5-dimethylthiazol-2-yl)-2,5-diphenyl tetrazolium bromide, 4′6-diamidino-2-phenylindole, bovine serum albumin, cytochrome C, EDTA, Griess reagent (modified), NADH, sodium selenate, thiobarbituric acid, trichloroacetic acid and xanthine were purchased from Sigma-Aldrich (Munich, Germany). The 1,2-dioleoyl-sn-glycero-3-phosphocholine (DOPC) and 1,2-dioleoyl-sn-glycero-3-phospho-(1′-rac-glycerol) (DOPG) were obtained from Avanti© Polar Lipids (Alabaster, AL, USA). The chloroform, dimethyl sulfoxide and pyruvic acid were from POCH (Gliwice, Poland). The Cellular ROS/Superoxide Detection Assay Kit was purchased from Abcam (Cambridge, UK).

### Cell cultures

The human histiocytic lymphoma cell line U-937 (ATCC) was cultured in a suspension in RPMI 1640 containing 5% fetal bovine serum and penicillin (100 U/ml) and streptomycin (0.1 mg/ml). Solutions of ZEN at concentrations of 1–300 μmol/l, Na_2_SeO_4_ (hereinafter referred to as Se) at concentrations of 0.5–30 μmol/l and EBR at concentrations of 0.1–100 nmol/l were tested. Based on the experiments of cell viability, the concentrations of ZEN (30 μmol/l), Se (2.5 μmol/l) and EBR (0.005 μmol/l) were selected.

### Cell viability assay

Cell viability was assessed using a colourimetric MTT ((3-(4,5-dimethylthiazol-2-yl)-2,5-diphenyl tetrazolium bromide) assay. Cells were cultured in 96-well plates with 0.2 × 10^6^ cells per well at a volume of 0.2 ml/well. After 24 h of exposing the cells to selected factors, 50 μL of a MTT solution at a concentration of 5 mg/l was added to each well and left for 2 h at 37 °C. Next, the entire volume of the wells was transferred into Eppendorf tubes (1.5 ml) and then, 0.4 mL dimethyl sulfoxide (DMSO) was added to the Eppendorf tubes. After 5 min, the solutions were centrifuged and the absorbance of the supernatants was read at a wavelength of 570 nm on an Epoch microplate reader (BioTek Instruments, Winooski, VT, USA).

### DAPI staining

In order to investigate the fragmentation of nuclei, 1 × 10^6^ cells in a 0.5-ml suspension were treated with 30 μmol/l ZEN for 24 h with or without Se (2.5 μmol/l) or EBR (5 nmol/l) and the suspensions were prepared on slides and then stained with 1 μg/ml 4′6-diamidino-2-phenylindole (DAPI) (DAPI-DNA complex Ex = 364 nm; Em = 454 nm). The morphology of a nucleus was observed using a Canon EOS 60D camera and a Delta IB-10 optical microscope set with a U filter (420 nm).

### Nitric oxide production

The cells were cultured in 24-well plates with 1 × 10^6^ cells per well at a volume of 0.5 ml/well. The cells were treated with the selected factors, adjusted to a final volume of a suspension equal to 0.5 ml and kept for 24 h. After the treatment, the supernatants were collected, centrifuged (1000×*g*, 5 min) and stored at − 20 °C. The nitric oxide (NO) production from the treated cells was quantified spectrophotometrically using a Griess reagent (modified) (Sigma-Aldrich, Munich, Germany). The absorbance was measured at 540 nm and the nitrite concentration was determined using a calibration curve.

### Cell membrane damage

A lactate dehydrogenase (LDH) assay was used as the marker of the entire cell membrane. The LDH activity was measured indirectly spectrophotometrically using a Thermo Scientific UV-Vis spectrophotometer (Waltham, MA, USA). This test is based on the ability of LDH to convert lactic acid into pyruvic acid and vice versa. LDH occurs in the cytoplasm of all cells and when the cell membrane is damaged, it is released into a culture medium. Pyruvic acid reacts with 2,4-dinitrophenylhydrazine, which leads to the formation of a coloured hydrazone. The amount of colour that is formed is proportional to the number of lysed cells. The cells were cultured in 24-well plates with silver nanoparticles with 0.3 × 10^6^ U-937 cells. After the treatment, the samples were collected and centrifuged (1000×*g*, 5 min, MPW-351RH) (MPW Med. Instruments, Warsaw. Poland). One hundred microliters of supernatant was added to the tubes containing 0.5 ml of 0.75 mmol/l pyruvate and 10 μl NADH (140 μmol/l) (heated at 37 °C for 10 min) and incubated for 30 min at 37 °C. Then, 0.5 ml of 2,4-dinitrophenylhydrazine was added. After 1 h, the absorbance was measured at 450 nm.

### Determination of the MDA concentration

Membrane lipid peroxidation was estimated using a thiobarbituric acid (TBA) reaction with malondialdehyde (MDA). The cells were cultured in 24-well plates with 2 × 10^6^ U-937 cells per well at a volume of 0.5 ml. After the treatment, the samples were collected and centrifuged (1000×*g*, 5 min). Vortex and 0.5 ml of 0.5% trichloroacetic acid (TCA) were added to the pellets, for 1 min and then lysed by sonicating for 5 min. After centrifugation at 10,000×*g* for 10 min, 0.4 ml of the supernatant was added to the 1.25 ml 20% TCA with 0.5% TBA and heated in a dry thermoblock (100 °C) for 30 min. After cooling, the absorbance was measured at 532 nm and corrected for any non-specific background by subtracting the absorbance at 600 nm. The MDA concentration was calculated using the molar excitation coefficient of 155 mmol/cm.

### Cellular ROS/superoxide radicals assay

Free radicals and other reactive species were detected using a Cellular ROS/Superoxide Detection Assay Kit (Abcam, Cambridge, UK). In a 0.5 ml suspension, 1 × 10^6^ cells/ml were incubated with 30 μmol/l ZEN for 24 h with or without Se (2.5 μmol/l) or EBR (0.005 μmol/l) and with a ROS/Superoxide Detection Mix for 30 min at 37 °C. The positive control was incubated with 0.2 mmol/l pyocyanine. After washing, superoxide (red) and oxidative stress (ROS, green)-positive cells were observed using a Canon EOS 60D camera (Tokyo, Japan) and a Delta IB-10 optical microscope set (Poland) with a B (520 nm) and G (590 nm) filter and fluorescence set (× 200 magnification).

### Superoxide dismutase assay

Total superoxide dismutase activity (SOD; EC 1.15.11) was assayed according to the spectrophotometric cytochrome method. Of cells, 1 × 10^6^ was homogenised in a 0.05 mol/l phosphate buffer (pH 7.2) containing 0.1 mmol/l EDTA and 0.1% bovine serum albumin. The homogenate was centrifuged for 10 min. at 10,000×*g*. The reaction mixture consisted of a phosphate buffer (pH 7.2), 0.1 mmol/l EDTA, 0.1 mmol/l cytochrome C and 0.1 mmol/l xanthine. Next, xanthine oxidase and supernatant were added. The absorbance was measured for 2 min at λ = 550 nm. It was expected that a unit of activity (one unit, one unit of cytochrome) would correspond to the amount of enzyme that caused a 50% inhibition of the reduction of cytochrome C at 25 °C. All of the measurements were carried out using a Thermo Scientific UV-Vis spectrophotometer (Waltham, MA, USA). The activity of SOD was expressed relative to the protein content in the supernatant. Protein was assessed by the method of Bradford (1976) using BSA as the protein standard.

### Langmuir technique for measuring the model membrane structure

The experiments were performed using the Langmuir technique (Minitrough, KSV, Espoo, Finland) to assess the physicochemical parameters of the lipid monolayers (Rudolphi-Skórska et al. 2016). Surface pressure (π) was detected using a Platinum Wilhelmy plate connected to an electrobalance with an accuracy of ± 0.1 mN/m. The monolayers were prepared by spreading chloroform solutions of the DOPC/DOPG mixture at a molar ratio of 2:1 on the surface of water or with a Se (2.5 μmol/l) and EBR (0.005 μmol/l) aqueous solution as the subphase. The subphase was redistilled water, which had been purified using a Milli-Q system, with a specific resistance of above 18.2 MQ cm^−1^. ZEN (30 μmol/l) was added directly into the water phase or into the water with selenate ions. All of the experiments were performed at 25 °C. The monolayers were compressed at a rate of 3.5–4.6 Å^2^/molecule × min, and the experiments were repeated three or four times to ensure a high reproducibility of the obtained isotherms to ± 0.1–0.3 Å^2^. The dependence of surface pressure versus the area per lipid molecule (*π*–*A*) was the basis for calculating the parameters of the lipid monolayer structure: A_lim_—the minimum area occupied by a single molecule in a fully packed layer and π_coll_—pressure at which a layer collapses and C_s_^−1^—static compression representing the mechanical resistance against the layer compression that provides information on the stability and fluidity of a layer.

### Electrokinetic potential of the model membranes

Liposomes were prepared according to the method described in detail by Filek et al. (2002) and Rudolphi-Skórska et al. (2018). The lipid mixture (DOPC+DOPG; 2:1, mol/mol) dissolved in chloroform, covering the wall with a round bottom glass tube in a stream of argon. The dried lipid film was hydrated with water (control—0) and vortexed. In the first experiment, ZEN solutions at 10, 20, 40 and 100 μmol/l were added to the media with liposomes and in the second, the liposomes were introduced into the media with ZEN (30 μmol/l), Se (2.5 μmol/l), EBR (0.005 μmol/l) and the mixture of ZEN with Se and EBR. The electrokinetic potential was calculated from the electrophoretic mobilities using the dynamic light scattering (DLS) method with a Malvern Zetasizer Nano ZS (Malvern Analytical Ltd., Malvern, UK). The mobility values were converted into electrokinetic potentials using the Smoluchowski equation.

### Statistical analysis

Data are presented as the mean ± SE. The experiments were repeated at least three times, and each experiment included at least three individual treatments. Data from the various treatments were statistically analysed using the SAS ANOVA procedure, and the mean comparisons were performed using Duncan’s test from PC SAS 8.0. Differences of *p* ≤ 0.05 were considered to be significant.

## Results

**Fig. 1:**
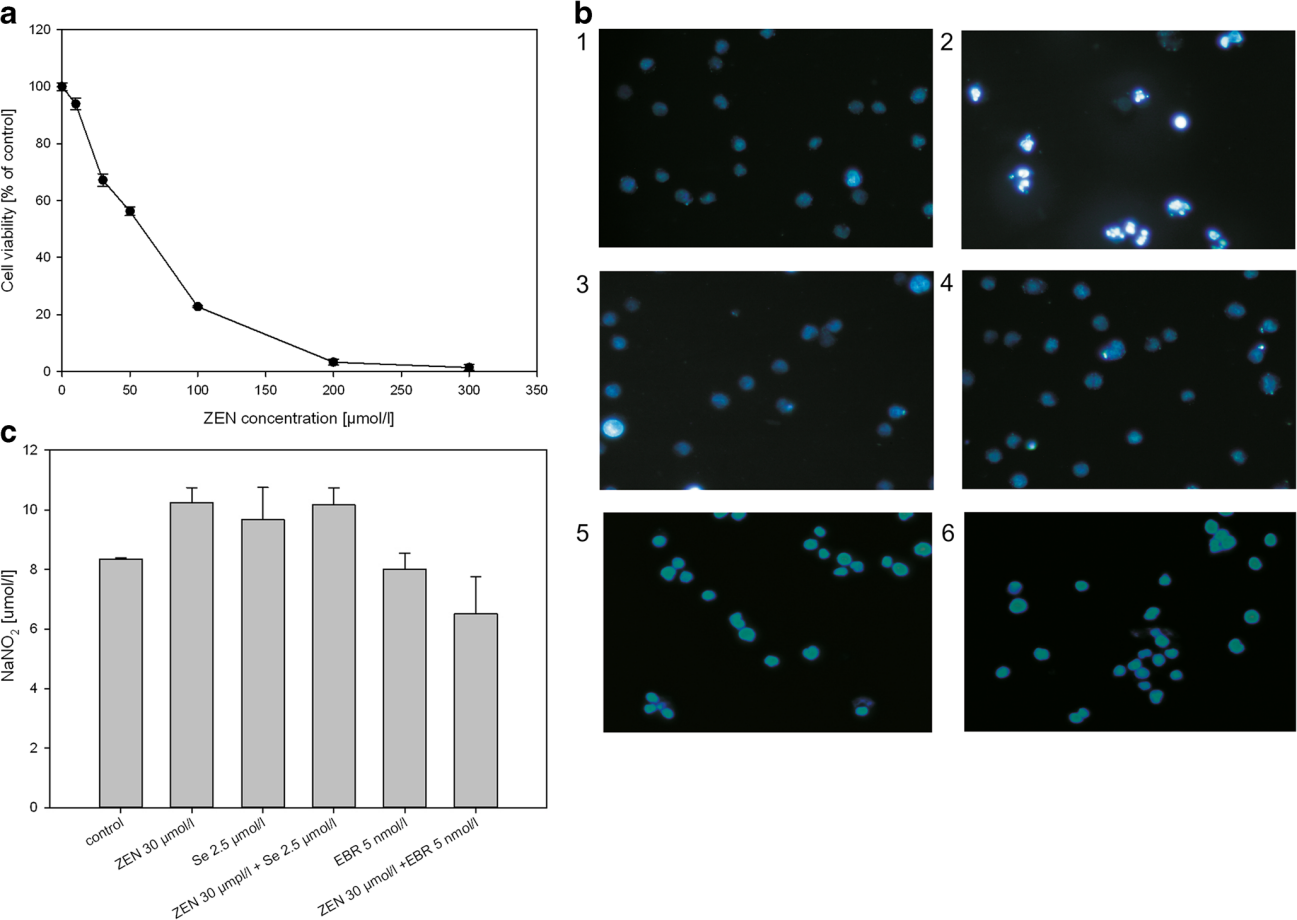
Viability of U-937 cells in media with ZEN (**a**); the observation of the fragmentation of the nuclei (**b**) of the untreated U-937 cells (1), cells treated with 30 μmol/l ZEN for 24 h (2), cells treated with Se (2.5 μmol/l) (3) or cells treated with EBR (0.005 μmol/l) (4), with 30 μmol/l ZEN and with Se (2.5 μmol/l) (5), with 30 μmol/l ZEN and with EBR (0.005 μmol/l) (6), analysed using DAPI-standing; induction of inflammation in U-937 cells after a 24-h incubation with ZEN indicated by the secretion of nitric oxide (**c**)

The cytotoxicity of ZEN was dependent on the amounts of this substance in the media and increased when the concentration of this mycotoxin was raised—at above 200 μmol/l of ZEN, all of the cells were dead (Fig. 1a). Relatively low concentrations of Se and EBR were selected based on previous experiments, which were connected with their protective activity in a ZEN-stress action. For Se, in the range of studied concentrations (0.5 to 30 μmol /l), the decrease in cell viability was only about 4% (at more than 10 μmol /l), relative to the control and for EBR, it was about 15% (at more than 20 nmol /l) compared to the control (results not shown). Thus, the following concentrations were selected to study the effect of these substances on cell viability: ZEN at 30 μmol/l, Se at 2.5 μmol/l and EBR at 0.005 μmol/l.

Microscopic observations (DAPI test) showed that in the presence of ZEN, the number of damaged cells significantly increased (compared to the control) (Fig. 1b). More segmented cell nuclei indicated that their nucleus was significantly damaged. The addition of Se and EBR in the mixture with ZEN reduced the number of damaged cell nuclei.

An analysis of the effect of the tested substances on the initiation of cell inflammation showed that the addition of ZEN and ZEN + Se resulted in an increase of the nitric oxide secretions relative to the control, while in the case of EBR (both, alone and in a mixture with ZEN), the opposite reaction (a decrease in the nitric oxide content in the culture medium) was observed (Fig. 1c).

**Fig. 2:**
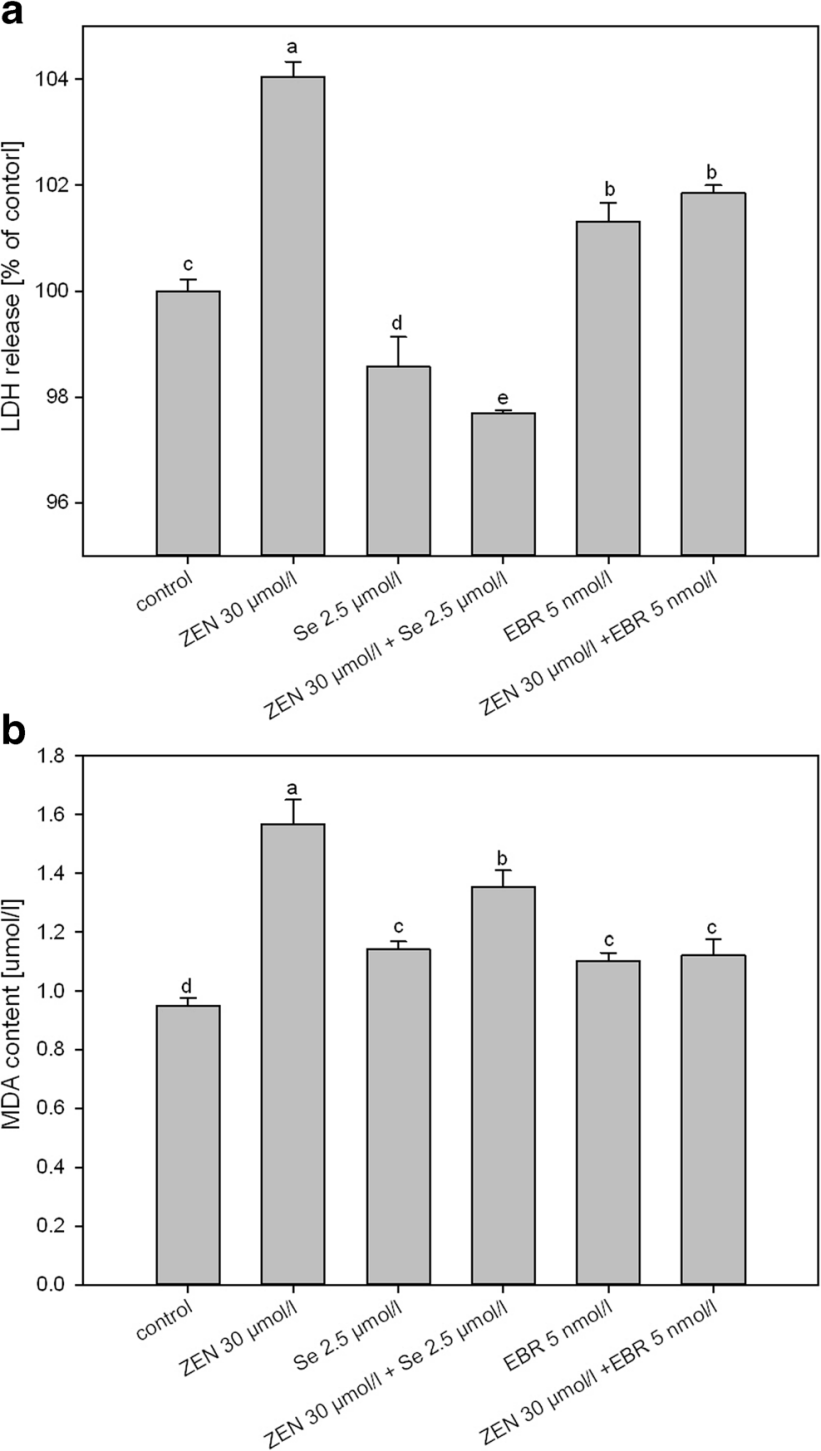
Membrane damage (**a**) determined via an LDH assay and the extent of membrane lipid peroxidation (**b**) expressed as the MDA content in the U-937 cells after 24 h of ZEN treatment

The study of membrane damage, which was measured using LDH and MDA tests, indicated an increase in the content of these substances under the influence of ZEN when it was added alone (Figs. 2a, b). In the LDH test, the Se ions stimulated a decrease, while EBR intensified the level of LDH compared to the control. When they were added together with ZEN, similar changes were observed in this test as in the case of separate applications. For MDA analysis, when both Se and EBR were applied with ZEN, they induced a decrease in the MDA concentration (compared to ZEN); however, when they were present alone, they increased the amounts of this indicator slightly.

**Fig. 3:**
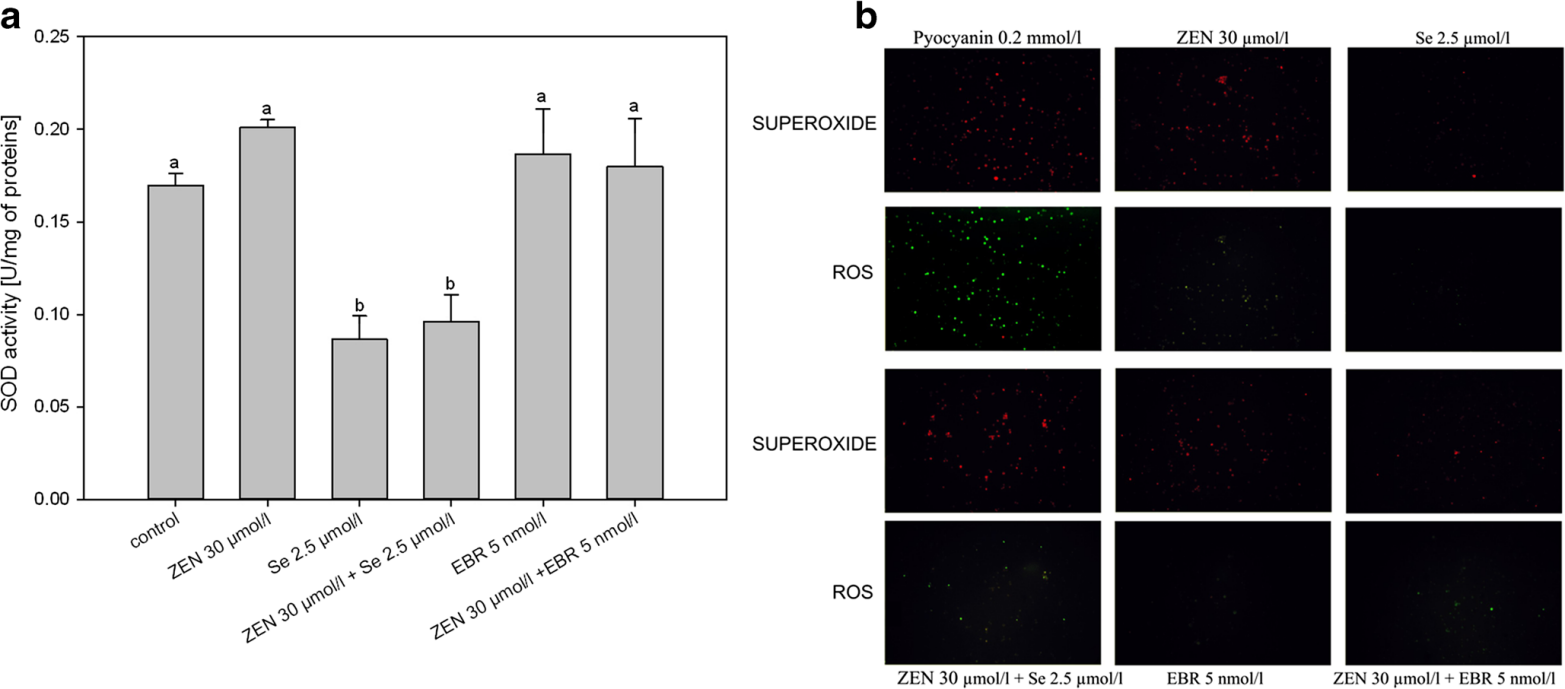
Effect of ZEN on the SOD activity (**a**) and cellular ROS/Superoxide production by U-937 cells after treatment with ZEN with or without Se and EBR compared to the positive control (cells treated with pyocyanine). General oxidative stress was observed in the green channel, while superoxide production was detected in the red channel (**b**)

The generation of ROS under the influence of ZEN was demonstrated in the measurements of the activity of the antioxidative enzymes (an increase in the SOD activity) (Fig. 3a) as well as by microscopic observations (Fig. 3b). The addition of Se reduced the SOD activity (for both: alone and in mixture with ZEN) significantly, while EBR did not induce significant SOD changes compared to the control (Fig. 3a). Analysis of the microscopic images enabled the superoxide radicals (orange channel) in the general pool of ROS (the green channel) to be characterised (Fig. 3b). The presence of ZEN increased both the total amount of ROS and, in particular, the level of superoxide radicals. When selenium and EBR were added individually, they did not affect the presence of ROS; only EBR initiated the creation of superoxide radicals. When these substances were mixed with ZEN, there was a significant decrease in the amount of ROS (compared to ZEN). EBR affects the total blocking of all of the ROS, while in the ZEN + Se mixtures, only small amounts of superoxide radicals were found.

**Fig. 4:**
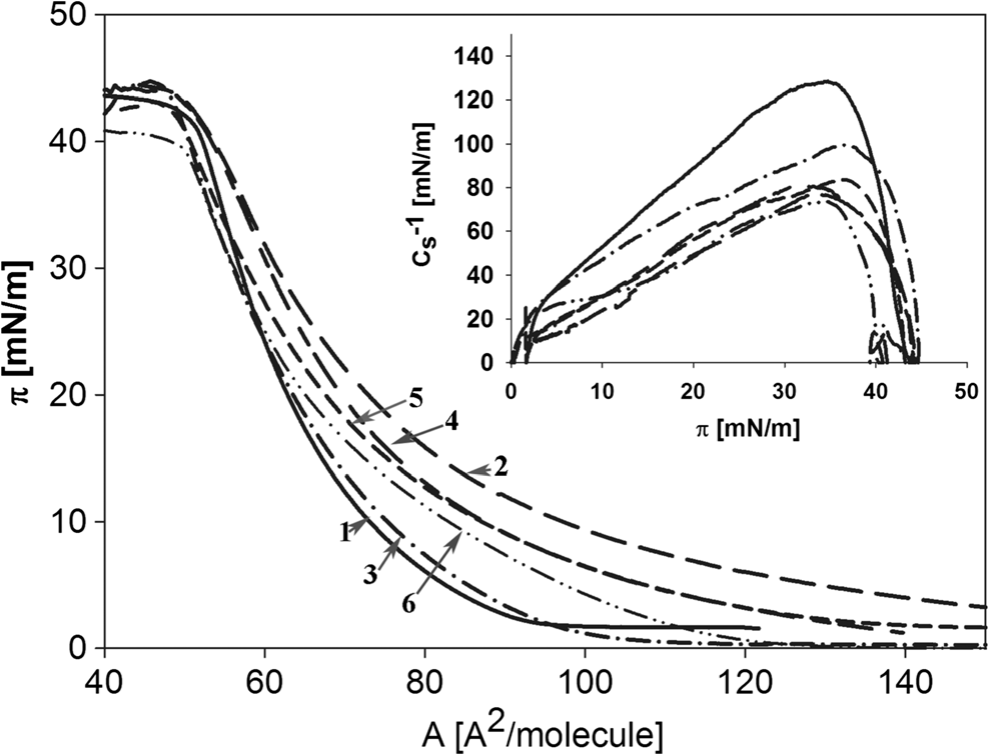
Examples of the surface pressure (*π*) versus area per molecule (**a**) isotherms obtained for the DOPC/DOPG mixture on water (1), 30 μmol/l ZEN (2), 2.5 μmol/l Se (3), ZEN + Se (4), EBR (0.005 μmol/l) (5) and ZEN + EBR (6). Inset: C_s_^−1^ versus π for the same systems

In model studies, the effect of the studied substances was observed on the lipid layers that had been built from 1,2-dioleoyl-sn-glycero-3-phosphocholine (DOPC), which is a lipid that is found in the largest quantities in membranes (Vance and Vance, 2008) and 1,2-dioleoyl-sn-glycero-3-phospho-(1′-rac-glycerol) (DOPG), which brings a negative charge to the lipid layer that is appropriate to the actual charge that occurs on the surface of the tested cell lines (based on the electrokinetic potential of U-937 cells and liposomes—data described later). Lipid monolayers of DOPC+DOPG (2:1, mol/mol) that formed on the water media to which the examined substances were added are shown in Fig. 4 as the dependence of the surface pressure (π) versus the area of the molecules (A) and the monolayer compressibility (C_s_^−1^) versus π (Fig. 4, insert). The physicochemical parameters of the membrane structures were calculated based on these dependences (Table 1). The presence of ZEN in the water media resulted in an increase in the surface that was occupied by the individual molecules in the monolayer (A_lim_) and a decrease in the pressure and lipid compressibility (C_s_^−1^) compared to the control (water). Se and EBR (separately) also increased the A_lim_, although to a lesser extent than those of ZEN. In the mixture with ZEN, there were greater values of A_lim_ than for this substance alone, especially in the case of Se. There was a similar tendency for the other parameters—a decrease in their mixture with ZEN, compared to the values that were obtained for Se and EBR alone as well as an increase compared to ZEN.

**Table 1:** Physiochemical parameters of the DOPC+DOPG (2:1, mol/mol) monolayers that were spread on the water subphase (control) or the subphase with ZEN (30 μmol/l), Se (2.5 μmol/l), EBR (0.005 μmol/l) and the mixture of ZEN with Se and EBR solutions calculated based on the π/A isotherms. For more details, see the “Material and methods” section. Values are the average ± SE. Different letters indicate statistically significant differences between the treatments (*P* < 0.05)

Monolayer system	A_lim_ [Å^2^]	Π_coll_ [mN/m]	C_s_^−1^ [mN/m]
Control	66.5 ± 0.1f	41.0 ± 0.5b	128.4 ± 0.5a
ZEN	84.9 ± 0.3a	40.2 ± 0.3c	77.7 ± 0.8d
Se	75.1 ± 0.2e	41.9 ± 0.4a	99.4 ± 0.7b
ZEN + Se	81.4 ± 0.4d	40.7 ± 0.2b	81.3 ± 0.8d
EBR	80.2 ± 0.2c	41.6 ± 0.4a	83.9 ± 0.6c
ZEN + EBR	80.9 ± 0.3b	40.9 ± 0.3b	78.8 ± 1.2d

The values of the electrokinetic potential of the liposomes that were obtained from the studied lipids depended on the concentration of ZEN in the tested media and increased at about 8 mV when the concentration of this toxin was increased from 10 to 100 μmol/l (Table 2). The presence of selenium in the media resulted in a decrease, while the presence of EBR resulted in an increase in the values of the electrokinetic potential of the liposomes compared to the values under the control conditions (0). The mixtures of ZEN + Se caused the electrokinetic potential to decrease (compared to those that were registered for ZEN alone at 30 μmol/l), whereas for the ZEN + EBR mixture, there was an increase in the tendency of the potential values (Table 3).

**Table 2:** Electrokinetic potentials of the liposomes that were obtained from the DPPC+DPPG (2:1) in the control media (0) and the media with ZEN (at 10, 20, 40 and 100 μmol/l). The values represent the average of six replicates ± SE. Different letters indicate statistically significant differences between the treatments (*P* < 0.05)

Treatments (concentration) [μmol/l	Electrokinetic potential [mV]
ZEN (0)	− 43.4 ± 0.2a
ZEN (10)	− 42.7 ± 0.3b
ZEN (20)	− 42.2 ± 0.3c
ZEN (40)	− 37.6 ± 0.5d
ZEN (100)	− 34.7 ± 0.4e

**Table 3:** Electrokinetic potentials of the liposomes that were obtained from the DPPC+DPPG (2:1) in the media with ZEN (30 μmol/l), Se (2.5 μmol/l), EBR (0.005 μmol/l) and a mixture of ZEN with Se and EBR at the selected concentrations. T values represent the average of six replicates ± SE. Different letters indicate statistically significant differences between the treatments (*P* < 0.05)

Treatments (concentration) [μmol/l]	Electrokinetic potential [mV]
CONTROL	− 43.5 ± 0.3c
ZEN (30)	− 39.5 ± 0.2f
Se (2.5)	− 47.3 ± 0.3a
ZEN + Se (30 + 2.5)	− 44.7 ± 0.5b
EBR (0.005)	− 42.9 ± 0.3d
ZEN + EBR (30 + 0.005)	− 40.2 ± 0.4e

## Discussion

The studies of the human U-937 cells indicated that an IC_50_ (the half maximal inhibitory concentration) factor for these cells was 57.09 ± 2.27 μmol/l of ZEN. The same IC_50_ value for ZEN was found for LO2 hepatocyte cells by Ku et al. (2014). On the other hand, Vero, Caco-2, DOK or HepG2 cells that had been treated with ZEN exhibited features of classical apoptosis at lower ZEN concentrations of 10–40 μmol/l (Abid-Essefi et al., 2004; Ayed-Boussema et al. 2008). The IC_50_ factor indicates that 50% of the cells were severely damaged and that above this value, cells may be destroyed. Thus, in our studies of the protective effect of Se and EBR against the action of a toxin, a concentration of 30 μmol/l ZEN was selected so that the possible recovery action of these substances were more visible in a higher percentage of cells that had not been destroyed. It was found that both Se and EBR increased the cell viability, while it was decreased in the presence of ZEN.

The ZEN-cytotoxicity was confirmed by an analysis of the LDH leakage assay and the ZEN-induced increase in the extracellular LDH level, which correlated with the number of damaged U-937 cells. Similar observations of a loss of plasma membrane integrity were made for neuronal cells (Venkataramana et al. 2014).

Finding that ZEN stimulated an increase in the MDA content indicates the initiation of membrane lipid oxidation as an effect of the oxidative stress action (Tomita et al., 1990). Changes that result from the oxidative stress that was caused by ZEN were also found in HepG2 and Caco-2 cells (Hassen et al. 2007; Kouadio et al. 2005) as well as in plant cells (Filek et al. 2017; Kornaś et al. 2018). An increase in the ROS concentration, which is a direct indicator and stimulator of oxidative stress, was observed in the presented experiments and the protective effects of Se as well as EBR causing a decrease in the amount of ROS were also observed.

The inclusion of defensive mechanisms against an excess of ROS was demonstrated as an increase in the activity of the antioxidant enzymes. The different reactions of the tested protective substances indicate the activation of various stages of the defence mechanisms. The decrease in the amount of ROS in the presence of Se is associated with a decrease in the SOD activity, which is responsible for the peroxidation of the superoxide anion radicals. This was confirmed by the observation of the total disappearance of the superoxide radicals in the ZEN + Se media. The selenium atom is incorporated into selenocysteine and in this form, it is included into enzymes such as glutathione peroxidase and type I iodothyronine 5′-deiodinase (Rayman 2000). Thus, Se has a significant impact on reducing ROS production. While EBR acts by stimulating the overall antioxidant system, EBR is also involved in the removal of H_2_O_2_ as has been demonstrated in earlier studies (Janeczko 2016). Thus, it is probable that the small amounts of superoxide radicals, which were not fully eliminated in the presence of EBR, were observed in the present experiments.

A precise analysis of the effects of Se and EBR on the membrane of the examined cells was performed in model experiments. DOPC, which is the main lipid in human cells, has been used in many studies to explain the first steps of the mechanisms that are involved in the interactions between various chemicals and membranes (Mosca et al. 2011; Rudolphi-Skórska et al. 2017). In the presented experiments, the mixture of DOPC with DOPG mimicked the physicochemical properties of the native human U-937 cells, which was confirmed by the measurements of the electrokinetic potential of the model and natural cells. There was a significant increase in the A_lim_ value (by about 30% compared to the control), a decrease in π_coll_ and an almost 40% decrease in C_s_^−1^ (compression module that indicates a decrease in the stiffness of the layer) in the presence of ZEN, which confirms the possibility that this toxin may also be found in human cell membranes. Such an assumption was proposed in the studies of the ZEN interaction with model plant cells (Gzyl-Malcher et al. 2017). When ZEN is incorporated into the monolayers, it decreases their stability and stiffness. The presence of Se and EBR in the mixture with ZEN partially reversed the influence of ZEN on the physiochemical properties of membranes. The larger Se effect compared to EBR on the modification of the membrane structure that was observed in the present experiments may result from differences in the interactions of both of these substances with the membranes. As was demonstrated in previous experiments, the presence of Se influences the modification of the structure of the molecules in the hydrophilic layer of lipids (Gzyl-Malcher et al. 2017), while EBR can also penetrate the hydrophobic part (such as ZEN and other hydrophilic-hydrophobic compounds) (Filek et al. 2018).

Based on the presented experiments, it can be concluded that ZEN exhibits cytotoxic activity against human U-937 cells with an IC50 equal to 57.09 ± 2.27 μmol/l. The addition of Se at 2.5 μmol/l and EBR at 0.005 μmol/l protects against the cytotoxic action of ZEN. Their protective mechanism is related to decreasing the production of ROS, which is stimulated in the presence of ZEN. Se reduces the ROS excess and thus prevents membrane lipid peroxidation and damage to the cells (measured as a decrease in LDH leakage), while the effect of EBR is based on an enhancement of the ability of cells to cope with the consequences of oxidative stress. This study of model membranes enabled the physicochemical parameters of lipid structure of U-937 cells to be precisely described. It was found that the destabilising effect of ZEN on lipid organisation in the membranes was “reversed” by both of the studied substances but to a greater extent in the presence of Se.

## Abbreviations

BRs: Brassinosteroids
DLS: Dynamic light scattering
DMSO: Dimethyl sulfoxide
DOPC: 1,2-dioleoyl-sn-glycero-3-phosphocholine
DOPG: 1,2-dioleoyl-sn-glycero-3-phospho-(1′-rac-glycerol)
EBR: 24-epibrassinolide
GM-CSF: Granulocyte macrophage colony-stimulating factor
IFN-γ: γ-Type interferon
IL: Interleukin
iNOS: Nitric oxide synthase
LDH: Lactate dehydrogenase
MDA: Malondialdehyde
MTT: 3-(4,5-dimethylthiazol-2-yl)-2,5-diphenyl tetrazolium bromide
NBT: Nitroblue tetrazolium
PMA: Phorbol esters, *phorbol 12-myristate 13-acetate*
RA: *Retinoic acid*
ROS: Reactive oxygen species
SOD: Superoxide dismutase activity
TNF-α: Tumour necrosis factor α
TPA: 12-O-Tetradecanoylphorbol-13-acetate
VD3: Cholecalciferol, *vitamin D3*
ZEN: Zearalenone
